# Interplay of Mendelian and polygenic risk factors in Arab breast cancer patients

**DOI:** 10.1186/s13073-023-01220-4

**Published:** 2023-09-01

**Authors:** Mohammed Al-Jumaan, Hoyin Chu, Abdullah Alsulaiman, Sabrina Y. Camp, Seunghun Han, Riaz Gillani, Yousef Al Marzooq, Fatmah Almulhim, Chittibabu Vatte, Areej Al Nemer, Afnan Almuhanna, Eliezer M. Van Allen, Amein Al-Ali, Saud H. AlDubayan

**Affiliations:** 1 College of Medicine, Imam Abdulrahman bin Faisal University, Dammam, Saudi Arabia; 2 Department of Medical Oncology, Dana-Farber Cancer Institute, Harvard Medical School, Boston, MA USA; 3 Cancer Program, The Broad Institute of MIT and Harvard, Cambridge, MA USA; 4 Harvard Medical School, Boston, MA USA; 5 Department of Pediatric Oncology, Dana-Farber Cancer Institute, Boston, MA USA; 6 Department of Pediatrics, Harvard Medical School, Boston, MA USA; 7 Boston Children’s Hospital, Boston, MA USA; 8 Center for Cancer Genomics, Dana-Farber Cancer Institute, Boston, MA 02115 USA; 9 Division of Genetics, Brigham and Women’s Hospital, Boston, MA USA; 10 College of Medicine, King Saud bin Abdulaziz University for Health Sciences, Riyadh, Saudi Arabia

**Keywords:** Low-pass whole genome sequencing, Imputation, Polygenic risk score, Breast cancer, Arab population, Age of onset, Pathogenic variants

## Abstract

**Background:**

Breast cancer patients from the indigenous Arab population present much earlier than patients from Western countries and have traditionally been underrepresented in cancer genomics studies. The contribution of polygenic and Mendelian risk toward the earlier onset of breast cancer in the population remains elusive.

**Methods:**

We performed low-pass whole genome sequencing (lpWGS) and whole-exome sequencing (WES) from 220 female breast cancer patients unselected for positive family history from the indigenous Arab population. Using publicly available resources, we imputed population-specific variants and calculated breast cancer burden-sensitive polygenic risk scores (PRS). Variant pathogenicity was also evaluated on exome variants with high coverage.

**Results:**

Variants imputed from lpWGS showed high concordance with paired exome (median dosage correlation: 0.9459, Interquartile range: 0.9410–0.9490). After adjusting the PRS to the Arab population, we found significant associations between PRS performance in risk prediction and first-degree relative breast cancer history prediction (Spearman rho=0.43, *p* = 0.03), where breast cancer patients in the top PRS decile are 5.53 (95% CI 1.76–17.97, *p* = 0.003) times more likely also to have a first-degree relative diagnosed with breast cancer compared to those in the middle deciles. In addition, we found evidence for the genetic liability threshold model of breast cancer where among patients with a family history of breast cancer, pathogenic rare variant carriers had significantly lower PRS than non-carriers (*p* = 0.0205, Mann-Whitney *U* test) while for non-carriers every standard deviation increase in PRS corresponded to 4.52 years (95% CI 8.88–0.17, *p* = 0.042) earlier age of presentation.

**Conclusions:**

Overall, our study provides a framework to assess polygenic risk in an understudied population using lpWGS and identifies common variant risk as a factor independent of pathogenic variant carrier status for earlier age of onset of breast cancer among indigenous Arab breast cancer patients.

**Supplementary Information:**

The online version contains supplementary material available at 10.1186/s13073-023-01220-4.

## Background

Individuals from the Greater Middle Eastern (GME) regions are significantly underrepresented in genomic studies, with less than 0.01% of the samples in Genome-wide Association Studies (GWAS) Catalog [1] and less than 0.8% of the samples in the Genome Aggregation Database (gnomAD) [2] reporting GME origin [3]. Compared to their European counterpart, GME populations have roughly a doubled rate of recessive Mendelian disease [4] and the region is experiencing a growing burden of breast cancer [5], with the average breast cancer patients presenting a decade earlier compared to patients in Western countries [6]. While prior studies have attributed the lower age of onset to lower population median age and other environmental and cultural factors [5, 7], the contributions from hereditary risk factors specific to the GME populations remain unknown. As part of the effort to increase genomic representation from GME populations, recent progress has been made with the Qatar Genome Programme, which sequenced over 6000 Qatari subjects with diverse GME ancestry backgrounds and revealed significant differences in breast cancer polygenic risk score (PRS) distributions between cancer-free populations with different GME ancestry backgrounds [8]. However, it remains unclear whether elevated PRS would correlate with decreased age of onset at a significant magnitude and for which subgroup would information on PRS be potentially clinically relevant. Furthermore, PRS is known to underperform in non-European populations [9], and its ability to capture biologically plausible hereditary breast cancer risk in GME populations has yet to be tested. Therefore, finding methods to accurately capture the common variant risk in GME populations, as well as understanding its relation to other known genomic risk factors such as carrying pathogenic rare variants in cancer-predisposition genes, is crucial for a holistic view of the genomic risk in the GME population and can lead to better design of intervention strategies in a population facing growing genomic health disparities [10].

**Fig. 1:**
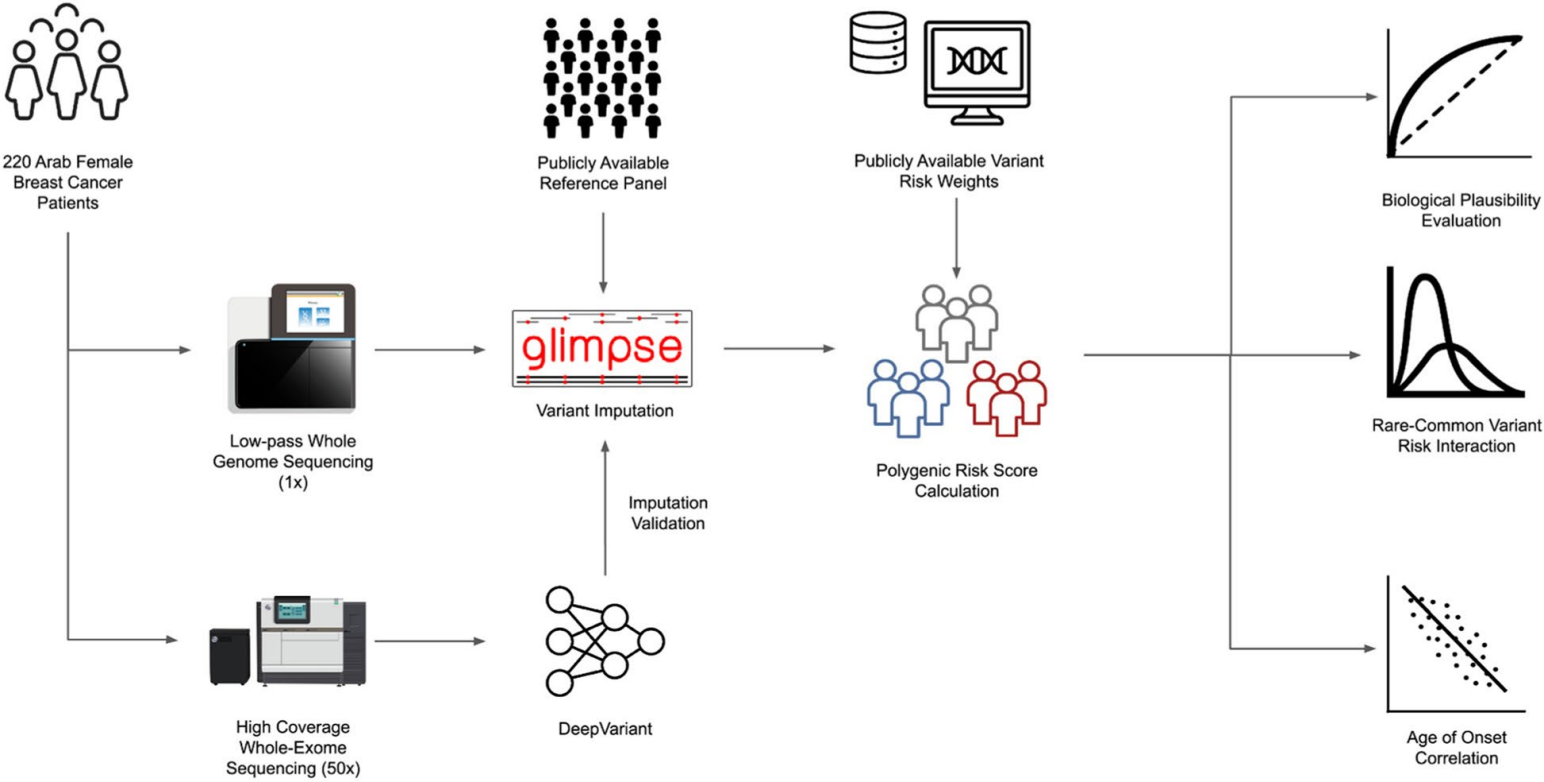
A graphical overview of the study. Low-pass whole genome sequencing (lpWGS) and high-coverage whole-exome sequencing (WES) were performed on blood samples collected from 220 indigenous Arab breast cancer patients. Variants were subsequently imputed and validated, and the polygenic risk scores (PRS) were calculated to facilitate downstream analysis of various clinical variables

Low-pass whole genome sequencing (lpWGS), or WGS with an average sequencing depth of around 1.0x, has recently been proposed as a cost-effective alternative data modality to study genetic architectures in understudied populations. Compared to the traditional genotyping arrays, lpWGS has reduced genetic variant ascertainment bias and has been shown to be sensitive to population-specific novel variants [11]. In addition, lpWGS has also been shown to outperform genotype arrays in imputation performance and statistical power [12–14]. Given these advantages, lpWGS appears as an attractive option to understand the polygenic architecture of breast cancer in GME populations, but its accuracy has yet to be systematically evaluated in a clinical setting. In this multicenter study, we collected blood samples from 220 female breast cancer patients from the indigenous Arab population who were not selected for positive family history or early age of onset and concurrently performed lpWGS and whole-exome sequencing (WES) on each sample. We imputed germline variants using publicly available reference panels and assessed their accuracy using the paired WES samples. Using the imputed variants, we calculated a population-adjusted PRS and discovered various interactions between polygenic risk and other clinical features such as family history, pathogenic rare variant burden, and age of onset (Fig. 1). Altogether, our investigation demonstrated an approach of using PRS to understand the polygenic risk landscape in an understudied population using patient data only without ancestry-matching cancer-free controls and highlighted the complementary role of rare and common risks in hereditary breast cancer in the indigenous Arab population.

## Methods

### Study participants

Blood samples from 220 female breast cancer patients from the indigenous Arab population unselected for early age of onset or family history of cancer were collected from 2 participating institutions in Eastern Saudi Arabia: King Fahd Hospital - Alhafouf and King Fahd University Hospital - Dammam. All individuals in this study consented to institutional review board-approved protocols that allowed for comprehensive genetic analysis of germline samples.

### Sequencing and library preparation

All samples (*n*=220) prepared for lpWGS had sufficient starting material (100 ng of double-stranded gDNA). Normalized DNA was fragmented (Covaris sonication) to 350 bp and then ligated to specific adapters during automated library preparation (Roche/KAPA, Hyper KK8504) using the Beckman FXp liquid handling robot. Libraries were pooled in equal volume and sequenced on an Illumina nano flow cell to estimate each library’s concentration based on the number of index reads per sample. Library construction is considered successful if the yield is larger than or equal to 250. All samples were successful. Libraries were normalized, pooled, and sequenced using Illumina platforms. Pooled samples were demultiplexed using the Picard tools version 1.130 [15].

For whole-exome sequencing (WES), a total amount of 1.0μg genomic DNA per sample was used as input material for the DNA sample preparation. Whole-exome capture libraries were generated using Agilent SureSelect Human All ExonV6 kit, and fragmentation was carried out by a hydrodynamic shearing system (Covaris, Massachusetts, USA) to generate 180–280bp fragments. Products were purified using the AMPure XP system (Beckman Coulter, Beverly, USA) and quantified using the Agilent high-sensitivity DNA assay on the Agilent Bioanalyzer 2100 system. The qualified libraries were fed into Illumina sequencers after pooling according to their effective concentration and expected data volume. All case samples had satisfactory effective read rates (> 97%) and error rates (< 0.03%) and are included in further analysis.

### Alignment

All raw sequencing data were uploaded to Terra (https://firecloud.terra.bio/), a collaborative cloud-computing platform utilized for genomic analyses, developed as part of the NCI Cloud Pilot program and supported by the Broad Institute [16]. Using Genome Analysis Toolkit (GATK) version 4.1.8.1 [17], all FASTQ files were first converted into unaligned Binary Alignment Map (uBAM) files, then aligned to the human reference genome b38 using BWA (version 0.7.15), as recommended by the GATK best practice workflows [18].

### Sequencing coverage

The average sequencing coverage of all lpWGS and WES samples was calculated using the GATK’s (version 3.7) tool “DepthofCoverage”. A sample-wide mean coverage of 0.1× was considered the minimum acceptable coverage for lpWGS, and a 15× average coverage over exon intervals was considered the minimum acceptable coverage for WES.

### Whole-exome variant calling

DeepVariant (version 1.0.0) [19], a deep learning-based variant calling method that has been demonstrated to have superior performance at detecting pathogenic variants compared to the standard joint-genotyping approach [20, 21], was used to call germline variants from WES data (docker image: gcr.io/deepvariant-docker/deepvariant:1.0.0). All variants annotated with “PASS” in the FILTER column of the VCF were deemed high quality. Variants passing QC from all samples were then merged into one VCF file using GATK’s (version 3.7) tool “CombineVariants”. Subsequently, the “vt” tool (version 3.13) was used on the cohort VCF file to normalize and decompose multiallelic variants.

### Functional and clinical annotation of germline variants

The cohort VCF file was annotated using Variant Effect Predictor (VEP, release 104.3) [22] with the publicly available GRCh38 cache file with a custom plug-in to include a recent “ClinVar” database release (accessed in June 2021). Using the tier criteria used by the Catalogue of Somatic Mutation in Cancer (COSMIC) [23], only variants in “germline tier 1” genes were considered. All detected variants were then classified into five categories: benign, likely benign, variants of unknown significance, likely pathogenic, and pathogenic, using the American College of Medical Genetics (ACMG) guidelines [24]. Moderately or highly penetrant variants classified as likely pathogenic or pathogenic are then collectively referred to as pathogenic variants (PV).

### Low-pass whole genome imputation

To obtain variant calls from lpWGS, GLIMPSE v1.1.1 [25] was used to perform genome-wide variant imputation. Following the recommended steps, the genome-wide genotype likelihood was first calculated on each sample using bcftools then separated into smaller genomic intervals before imputation. To maximize the number of variants imputed, we used Eagle v2.4.1 [26] to computationally phase the publicly available 1000 Genome [27] (1KG) WGS VCFs that were called using DeepVariant [28] (v1.0.0, GLnexus v1.2.7, GRCh38 reference), and used the output as the reference panels for imputation. After imputation was carried out on each genomic chunk, they were combined using the “GLIMPSE_ligate” command with default arguments, producing the final imputed VCF.

### Imputed variant quality control

To assess variant imputation accuracy and to select a proper filtering threshold, the concordance of exonic variants was calculated based on the intersection of variants called both by DeepVariant using WES data and imputed by GLIMPSE using lpWGS. The “INFO” score outputted by GLIMPSE, which is a value that ranges from 0 to 1 where 1 indicates high confidence in the variant call, is referred to as the imputation quality score. The linear transformation of the posterior genotype probabilities generated by imputation, which is a number ranging from 0 to 2 where a number close to 1 indicates confidence in a single alternate allele at the location while a number close to 2 reflects confidence in having 2 alternate alleles at the location, is referred to as variant dosage. Variants were binned based on minor allele frequency, and the correlation between variant dosage and the number of alternate alleles (0, 1, or 2) called by DeepVariant, referred to as dosage correlation, was calculated within each bin for every sample. Allele frequencies were calculated based on allele counts in the cohort.

### Relatedness inference

To control for confounding effects from related individuals, PLINK 1.9 [29] was first used to extract biallelic single-nucleotide polymorphisms (SNPs) from the merged WES VCF file. Subsequently, LDAK 5.2 [30] was used to compute a kinship matrix assuming the LDAK-Thin heritability model with a correlation squared threshold of 0.98 and window size of 100 kb, as recommended (https://dougspeed.com/calculate-kinships/). Samples were then removed until no pairs have a kinship value greater than 0.125. Five samples were removed after this step.

### Polygenic risk score calculation

To assess the clinical applicability of PRS, we adopted a similar PRS calculation methodology proposed by Hao L. et al. [31] and curated the initial sets of PRS weights from “CancerPRSWeb” [32], a repository that contains PRS coefficients for major cancer traits derived from multiple large population databases such as the UK BioBank (UKB) [33], Michigan Genomics Initiative (MGI) [34], and GWAS Catalog [1]. To pick PRS sets most relevant to breast cancer, we selected “Breast Cancer [Female]” as the cancer trait and manually curated 20 sets of non-subtype-specific weights which had validation performance in either MGI or UKB. The number of SNPs in the selected weights ranged from 79 to 1,120,410, and they were derived using various methods with different performances in UKB or MGI, as measured by area under the receiver-operator characteristic curve (AUC_population_). After downloading the associated weight file and metadata, SNPs with hg19 coordinates were lifted over to hg38 using the python liftover library [35] for downstream compatibility. For each set of PRS weights, we calculated the unadjusted raw PRS in PLINK 1.9 [29] by using the “--score” command with the “score-no-mean-imputation” option enabled.

### PRS population stratification adjustment

To obtain the genetic principal components (PCs) of every sample, we first merged the Arab breast cancer cohort WES VCF with the WES VCF from the 1000 Genomes Project [27]. The merged WES VCF was then loaded using Hail v0.2 [36] and filtered for variants with allele frequency > 0.05 and *p*-value greater than 1e−6 from the Hardy-Weinberg Equilibrium test. LD-pruning was then performed on passing variants with greater than 0.1 correlation within a 1 million base pair window. The Hail function “hwe_normalized_pca” was then applied to the resulting set of common variants (*n*=58,286), and the top 10 PCs were kept for further analysis.

To create a population-adjusted PRS, an ordinary least square model was fitted using the top 10 PCs as features with the raw PRS as the output variable. The difference between the predicted PRS and raw PRS was then standardized, creating the population-adjusted, residualized PRS. This process was then repeated for every set of PRS weights, and the CancerPRSWeb ID of the PRS weights with the highest performance at detecting breast cancer in first-degree relatives (*AUC*_*family*_) as well as any degree relatives (AUC_family-any_) was “PRSWEB_PHECODE174.1_Onco-iCOGS-Overall-BRCA_LASSOSUM_MGI_20200608.”

### Statistical analysis

Unless otherwise specified, all odds ratios, 95% confidence intervals, and *p*-values were computed based on the two-sided Fisher’s exact test, as implemented in the exact2x2 R package [37] with the argument “minimum likelihood correction.” Confidence intervals of the area under the receiver-operator characteristic curves (AUC) were calculated based on the formulation by J. Hanley and B. McNeil [38]. Statistical diagrams were visualized using Seaborn v0.11.2 [39]. Statistical models were constructed using the python package “statsmodel” [40]. The clinical characteristics table was constructed using the package “tableone” [41]. The effect of PRS standard deviation increase on the age of onset is estimated by fitting a generalized linear model (GLM) using the statsmodel package with standardized ancestry-adjusted PRS as the feature and age of presentation as the outcome. The coefficient of the PRS and its 95% confidence intervals as indicated by the GLM is then reported.

To evaluate the change in PRS AUC after removing pathogenic variant carriers from the cohort, the *p*-values were obtained by calculating the proportion of samples in which the AUC was lower after removing pathogenic variant carriers using 10,000 bootstrapped samples of the analysis cohort.

## Results

### Sample characteristics

**Fig. 2:**
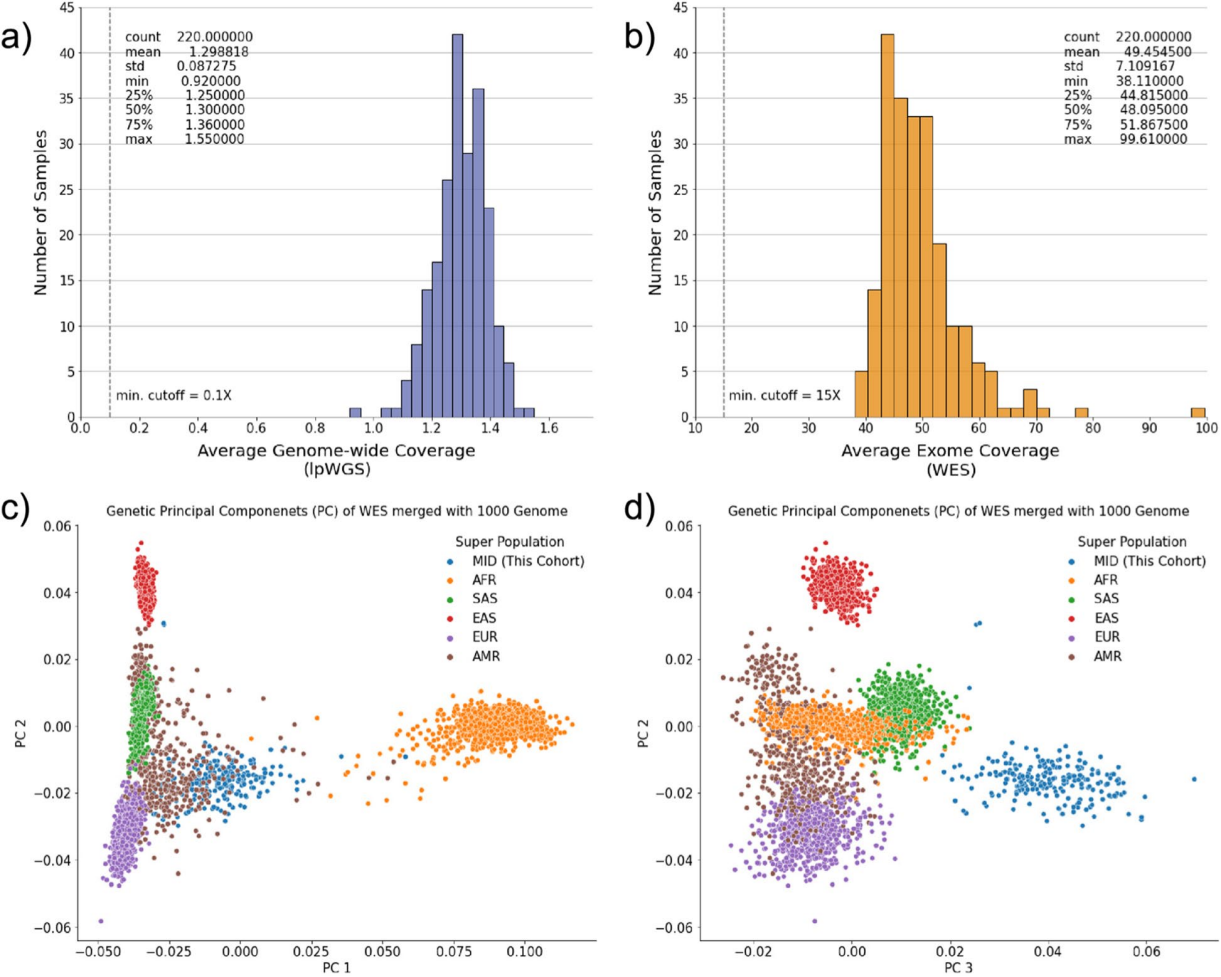
Sequencing metrics and sample characteristics of the cohort. **a** The average genome-wide coverage of lpWGS. **b** The average exome coverage of WES. **c, d** The first three genetic principal components based on the exomes merged with the 1000 Genomes data

All samples (*n*=220) met the minimum sequencing coverage cut-off, where the median genome-wide coverage for lpWGS was 1.3× (interquartile range [IQR] 1.25–1.36×, Fig. 2a) and median exome-wide coverage for WES was 48.1X (IQR 44.8–51.8×, Fig. 2b). After removing related individuals, defined as having a kinship coefficient above 0.125, a total of 215 female breast cancer patients of Middle Eastern ancestry were included in the final analysis (Methods). The WES variant calls were merged with variant calls of the 1000 Genomes Project [27], and the first 10 genetic principal components were calculated (Methods). As expected, due to the lack of Middle Eastern ancestry representation from the 1000 Genomes Project the PCs of the cohort form a cluster distinct from the rest of the samples (Fig. 2c, d). Among those whose clinical information was available, the mean age of presentation was 47.7 years (SD 10.1 years). The clinical characteristics of the breast cancer cases including histology and estrogen receptor status stratified by family cancer history status can be found in Additional File 1: Supplementary Table 1.

### lpWGS enables accurate imputation of low-frequency population-specific variants

**Fig. 3:**
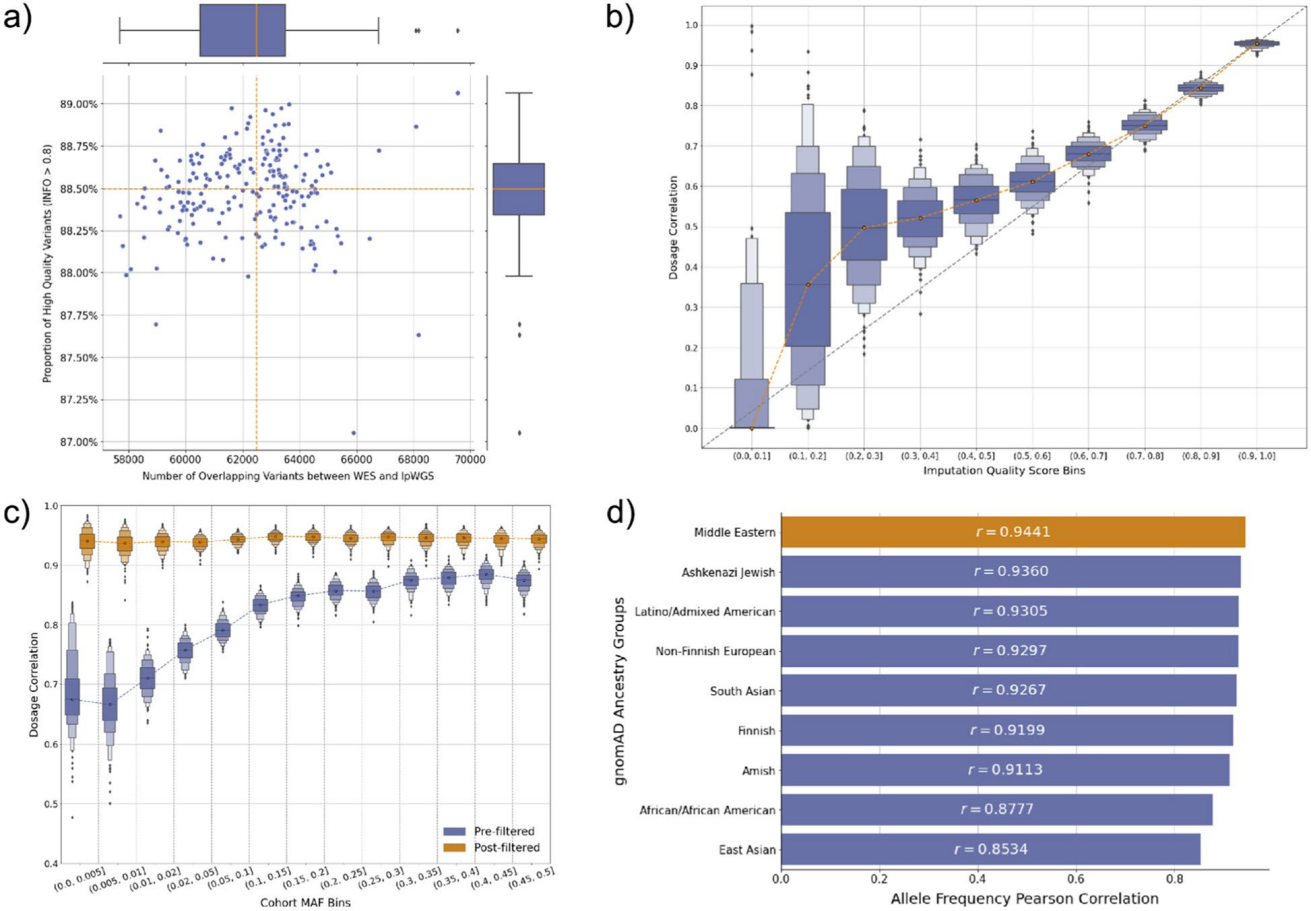
Imputation accuracy of lpWGS using high-coverage WES variants as ground truth. **a** The number of variants both imputed by lpWGS and called by high-coverage WES per sample and the proportion of these variants with imputation quality score (INFO) at least 0.8. **b** Boxen plot of the dosage correlation per sample grouped by imputation quality score intervals. Dosage correlation is defined as the correlation between the imputed variant dosage, which is a continuous value ranging from 0 to 2, and the number of alternate alleles called from WES. **c** The dosage correlation of lpWGS imputed variants grouped by cohort minor allele frequency before and after filtering out variants with imputation quality score below 0.8. We observe consistently strong performance after filtering regardless of MAF bins. **d** The Pearson correlation between the allele frequency of the imputed variants in our cohort versus their allele frequencies in gnomAD ancestry groups

To assess the quality of lpWGS-derived genotypes using publicly available reference panels, we systematically analyzed the imputation performance of lpWGS in the exome-regions using high-coverage WES-derived high-quality variants as the ground truth. The median number of intersecting variants both called by WES and imputed by lpWGS per sample was 62,495 (IQR 60,518–63,495), where at least 87.05% of the overlapping variants had imputation quality score greater than 0.8 across all samples (Fig. 3a). To assess the reliability of the imputation quality score in reflecting the true posterior probability of the imputed variant having the specified variant dosage, we grouped imputed variants into bins by their imputation quality scores and calculated the dosages correlation for each sample within each bin (Methods). We observed good correspondence between imputation quality scores and genotype called from WES where variants in the 0.8–0.9 imputation quality score bin have median dosage correlation in a similar range (0.8441, IQR 0.8355–0.8515) (Fig. 3b, Additional File 1: Supplementary Table S2). Collectively, the medians of dosage correlation per bin were highly positively correlated (Pearson correlation: 0.944, *p* < 0.001).

Next, we evaluated the impact of minor allele frequency (MAF) on variant imputation quality by stratifying the variants into MAF bins and calculating the dosage correlation within each bin. We found after filtering out variants with imputation quality scores below 0.8, the dosage correlations are consistently strong regardless of MAF (Fig. 3c, Additional File 1: Supplementary Table S3), and collectively, the median dosage correlation per sample after filtering was 0.9459 (IQR 0.9410–0.9490). As such, all variants with imputation quality score > 0.8 are included in downstream analysis without further filtering on MAF. To evaluate if the imputed variants are population-specific, we obtained the gnomAD [2] population allele frequencies for all imputed variants used for performance evaluation (*n*=284,601 variants) and calculated its Pearson correlation with the allele frequency in each of the gnomAD ancestry groups. As expected, we found our cohort’s variant minor allele frequencies to be the highest correlated with the Middle Eastern gnomAD ancestry group (Pearson correlation = 0.944, *p* < 0.001, Fig. 3d).

### Imputed variants enables calculation of breast cancer burden-sensitive polygenic risk score in the Arab population

**Fig. 4:**
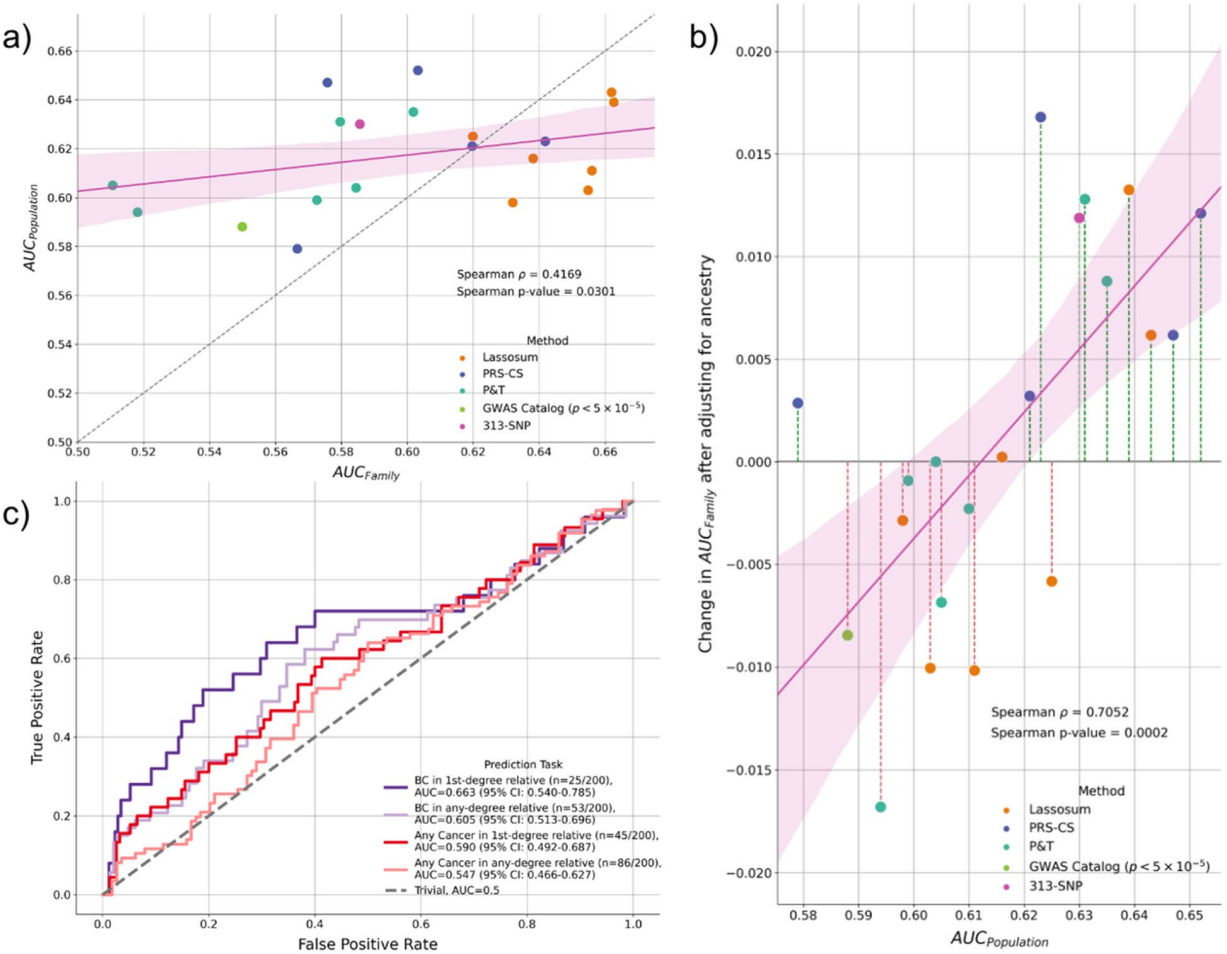
Evaluating the biological plausibility of the calculated PRS. **a** The Spearman correlation between the performance of PRS at detecting breast cancer in first-degree relatives (AUC_family_) vs. the reported performance of the PRS at detecting breast cancer patients in larger European populations from previous studies (AUC_population_). Method refers to the original method that was used to derive the weights for the PRS. **b** The original performance of the PRS (AUC_population_) plotted against the improvement in PRS AUC_family_ after the PRS is adjusted for population. The performance improvement is positively correlated with its original performance in the overall population suggesting the adjustment process was able to magnify burden effects while suppressing population stratification. **c** Evaluating the effectiveness of the best-performing PRS at predicting various cancer-related family histories. The PRS performs the best at predicting the presence of breast cancer in first-degree relatives and performance decreases as the relative degree increases and the cancer type become non-breast cancer-specific. BC: breast cancer

Using the high-quality imputed variants of Arab breast cancer patients, we adopted a PRS calculation pipeline similar to the one proposed by Hao et al. [31] and calculated 20 sets of breast cancer PRS for every sample using publicly available weights from “CancerPRSWeb” [32], a repository that contains PRS coefficients for major cancer traits derived from multiple large population databases, as well as the widely used 313 SNPs breast cancer PRS (Methods) [42]. To account for population stratification, each PRS was residualized against the top 10 genetic principal components and then standardized for subsequent analysis (Methods, Additional File 1: Supplementary Table S4). To evaluate each PRS’s ability to detect polygenic risk burden, we calculated the AUC of each PRS at the task of predicting patients with a self-reported family history of breast cancer at the first degree (AUC_family_). We found a positive correlation between the reported performance of the PRS from previous studies at detecting breast cancer patients in larger, mostly European populations (AUC_population_) and AUC_family_ (Spearman coefficient = 0.417, *p*-value = 0.0301) (Fig. 4a), suggesting the calculated PRS was able to detect similar breast cancer burden from family cancer history as well as in general population.

To evaluate the effect of population adjustment on PRS, we compared the difference in AUC_family_ before and after applying residualization. We found the original population performance of the PRS to be positively correlated with the improvement in AUC_Family_ after adjusting for ancestry (Spearman correlation 0.7052, *p*-value 0.0002) (Fig. 4b). That is, for PRS with a lower AUC_population_, population adjustment resulted in lower performance in AUC_family_, while for PRS with a higher AUC_population_, population adjustment resulted in higher performance in AUC_family_. This suggests the population adjustment process was able to mask population-specific signals from PRS with lower AUC_population_ while amplifying causal signals from PRS with high AUC_population_, which improves the power for our subsequent analysis.

Among the 21 sets of PRS for which the performance was evaluated (Additional File 1: Supplementary Table S4), the PRS with the highest AUC_family_ performance was chosen for downstream analysis (AUC_family_ 0.663, AUC_population_ 0.639, Number of SNPs 118,388). To further validate the biological plausibility of the calculated PRS, we evaluated its performance at identifying patients with a family history of breast cancer or other cancers at varying degrees. We found the performance of the PRS to be the strongest at identifying patients with first-degree relatives with breast cancer (AUC_family_ 0.663, 95%CI 0.540–0.785), and the performance decreased when higher-degree relatives with breast cancer were included (AUC_family-any_ 0.605, 95%CI 0.514–0.697) or when non-breast cancer was included (AUC 0.590, 95%CI 0.492–0.687) (Fig. 4c). As a negative control, age was not an effective predictor of family history of breast cancer (AUC_family_ 0.531, 95%CI 0.408–0.654) and the same performance ranking did not hold (Additional File 2: Fig. S1).

### Genetic liability threshold model in Arab familial breast cancers

**Fig. 5:**
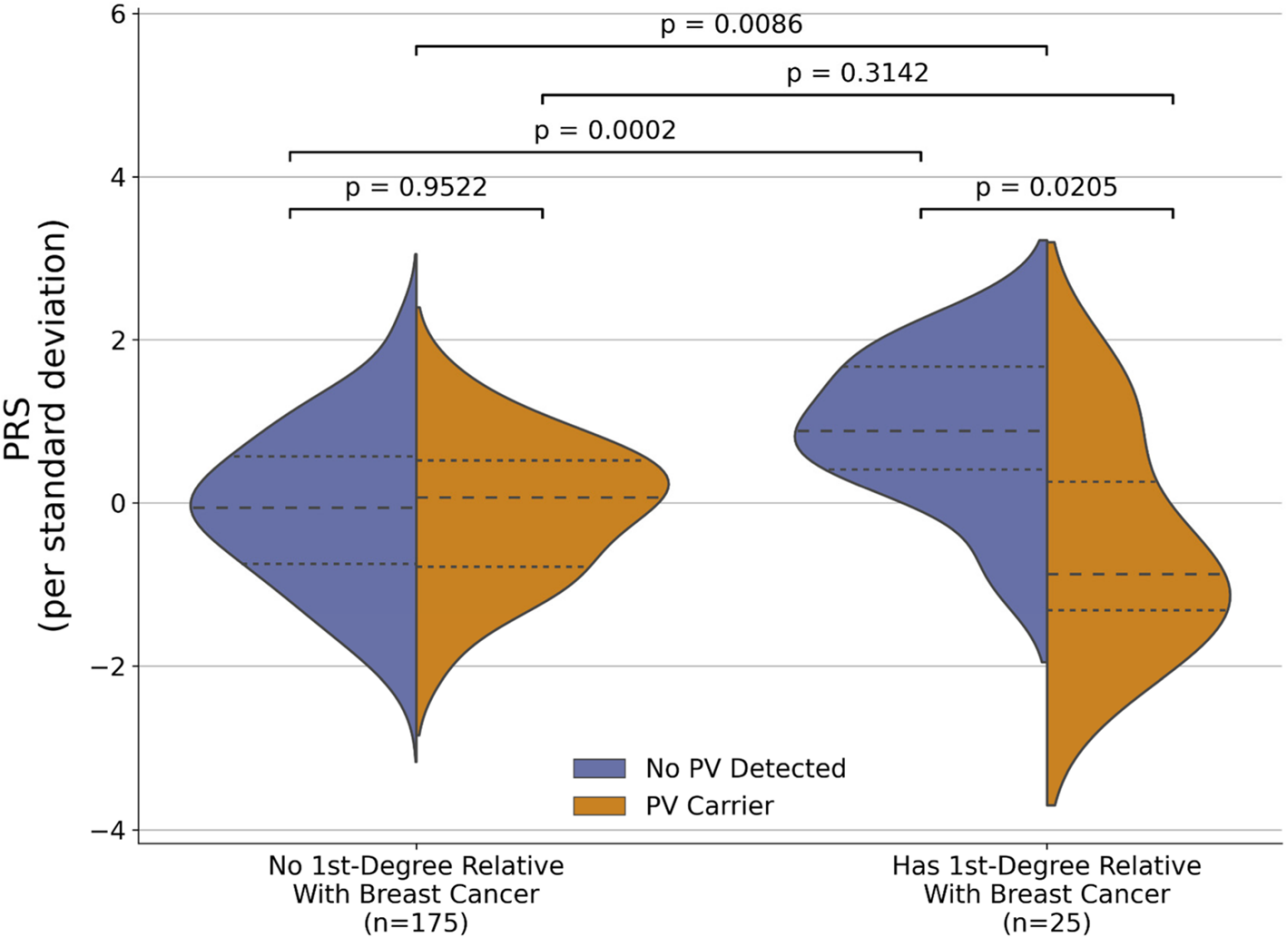
The interaction between rare pathogenic variant, PRS, and family breast cancer history in Arab patients with breast cancer. **a** Violin plots of the distributions of PRS between patients with or without first-degree relatives diagnosed with breast cancer, stratified by rare pathogenic variant carrier status (Patients with no first-degree relative with breast cancer: *n*=175, PV carriers: *n*=27, non-PV carriers: *n*=148. Patients with first-degree relatives with breast cancer: *n*=25, PV carriers: *n*=7, non-PV carriers: *n*=18). The dotted line indicates the first and third quartiles and the dashed line indicates the median

To understand the relative risk contribution of common variants and rare variants to hereditary breast cancer in Arab breast cancer patients, we next used WES data to identify 34 (15.81%) patients who carried a pathogenic variant (PV) in one of the known cancer-predisposition genes as outlined by the COSMIC database (Additional File 1: Supplementary Table S5, Additional File 2: Fig S2, Methods). Compared to patients with no first-degree relatives with breast cancer (*n*=175), Arab breast cancer patients who have first-degree relatives with breast cancer (*n*=25) had higher PRS (*p* = 0.0086, Mann-Whitney *U* test (M.W.U.)) (Fig. 5a) and were 2.13 times more likely to carry pathogenic variants (95% CI 0.81–5.59, *p* = 0.103). We found no significant difference in PRS distribution between PV carriers and non-carriers in patients with no first-degree relative with breast cancer (PV carrier: *n*=27, non-PV carrier: *n*=148, *p* = 0.946, M.W.U.), but among those who have a first-degree relative with breast cancer, non-PV carriers had significantly higher PRS than PV carriers (non-PV carrier: *n*=18, PV carrier: *n*=7, *p* = 0.0205, M.W.U.), suggesting a genetic liability threshold model of breast cancer where the threshold for breast cancer may be achieved through a combination of rare or common variant risk [43, 44]. In addition, among non-PV carrier patients, those with first-degree relatives with breast cancer have significantly higher PRS (*p* = 0.0002, M.W.U.) compared to those who do not (Fig. 5a). In contrast, no significant difference in PRS distributions was found among PV carriers based on first-degree relative breast cancer status (*p* = 0.3142, M.W.U.).

### Common variant risk is associated with earlier age of onset in PV-negative Arab breast cancer patients with family history

**Fig. 6:**
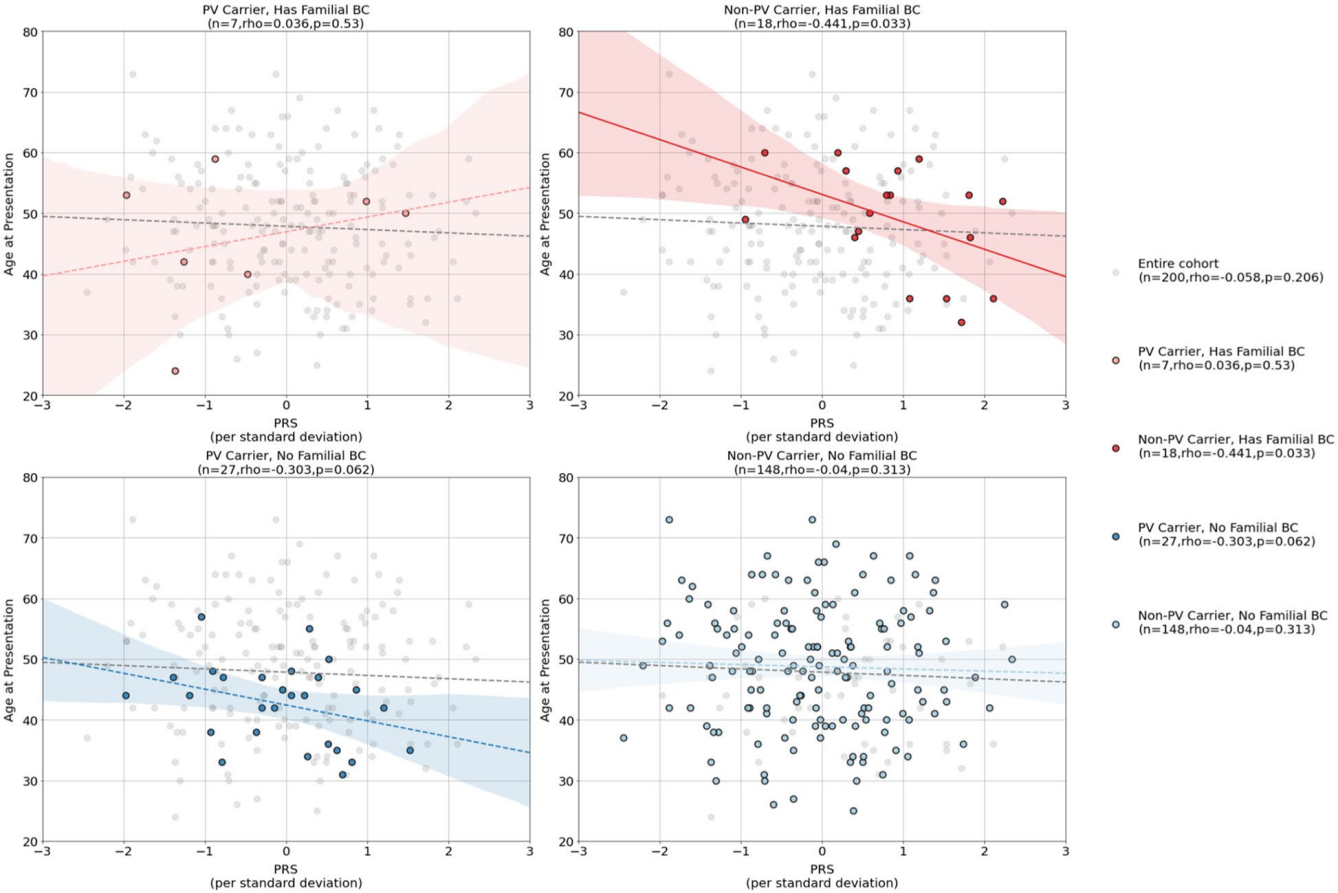
The interactions between age and polygenic risk score (PRS) conditioned on family breast cancer history (1st-degree relative) and pathogenic variant (PV) carrier status. The overall cohort had no significant correlation between age and PRS (gray line in all panels) but among patients with first-degree relatives diagnosed with breast cancer, age of onset is negatively correlated with PRS in patients with no detected pathogenic variants (top right panel). A similar trend can also be seen among PV carriers with no family history of breast cancer (bottom left panel)

Given the performance of PRS at detecting familial breast cancer risk, we next assessed whether elevated PRS was associated with an earlier age of onset. We found no statistically significant negative association between PRS and age in the overall cohort (rho=−0.05, *p* = 0.206). However, when conditioned on family history and PV carrier status, we found a statistically significant negative association between age of onset and PRS in patients with a first-degree relative with breast cancer but are negative for rare germline pathogenic variants (*n* = 18), (Spearman rho: −0.441, *p* = 0.033) (Fig. 6), where each standard deviation increase in PRS corresponded to 4.52 (95% CI 8.88–0.17, *p* = 0.042) years decrease in age of onset (intercept term 53.09 years, 95%CI 47.6–58.5, *p* < 0.001). A similar but weaker trend can be found in PV carriers without a first-degree relative with breast cancer, where each standard deviation increase in PRS corresponded to 2.61 (95% CI 0.33–5.56, *p* = 0.082) years earlier age of onset (intercept term: 42.43 years, 95%CI 40.0–44.9, *p* < 0.001, Fig. 6). To compare the effect of carrying a high common variant risk to carrying a pathogenic variant, we further assessed the age of onset distribution differences between PV carriers and non-carriers conditioned on family breast cancer history (Additional File 2: Fig. S3). We found that overall PV carriers present on average 5.5 years earlier compared to non-PV carriers (PV carrier mean age 43.3 years, non-PV carrier mean age 48.8 years, *p* = 0.0032, M.W.U.), where the difference in age distribution is mostly driven by PV carrier status in patients without a family history (*p* = 0.0026) and no significant difference in age distribution was found by PV carrier status within those with family breast cancer history (*p* = 0.56). Taken together, the results suggest common variant risk plays a prominent role in the earlier age of onset in hereditary breast cancer, especially in patients with a family history of breast cancer but negative in carrying pathogenic variants in known cancer-associated genes.

### PRS performance is influenced by rare pathogenic variant carrier status

**Fig. 7:**
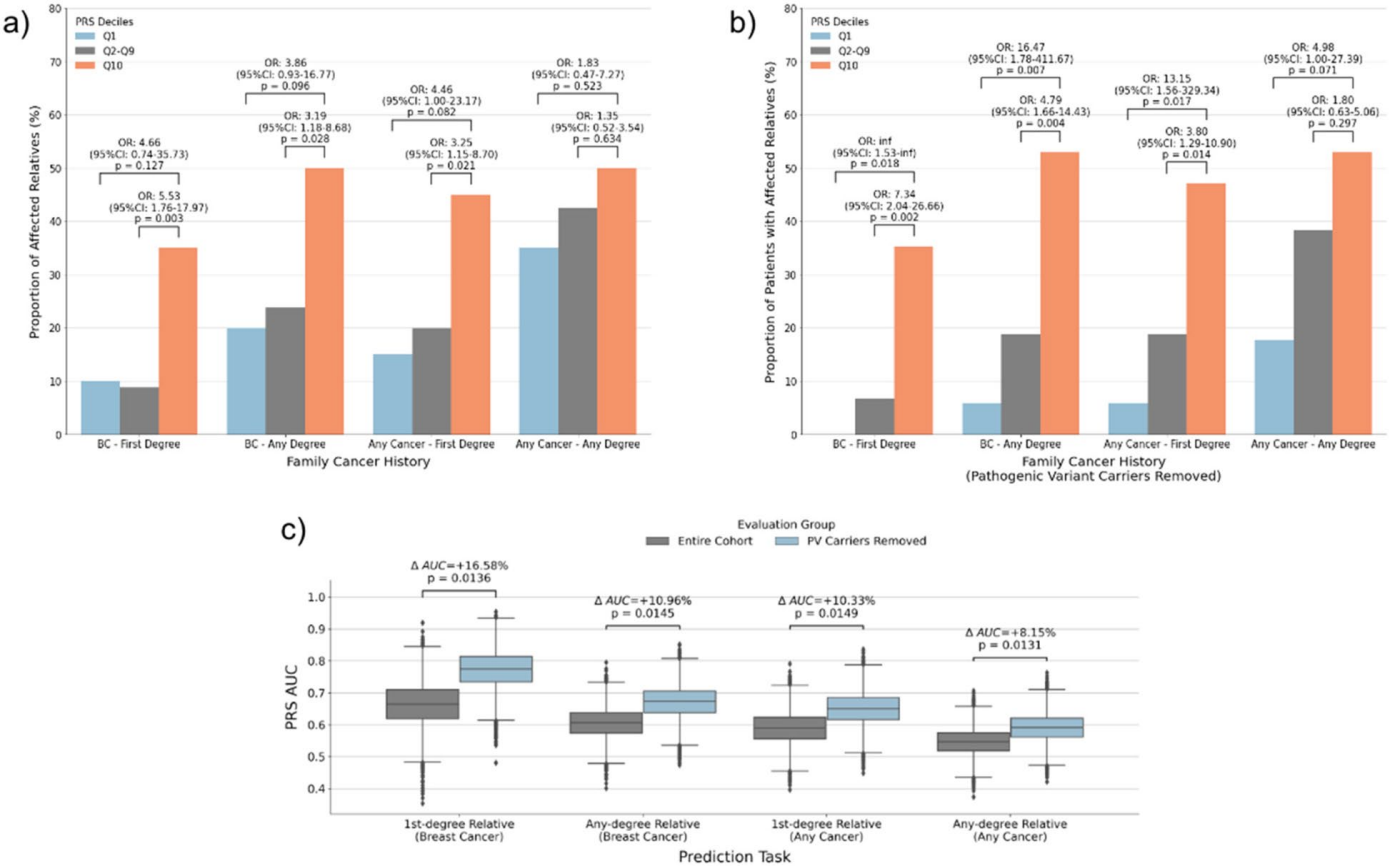
PRS Performance improves when pathogenic variants are accounted for. **a** The proportion of patients in different PRS deciles having a family history of breast cancer or other cancers. **b** The proportion of patients in different PRS deciles having a family history of breast cancer or other cancers after PV carriers are removed. **c** The performance of the PRS at detecting familial cancer risk before and after removing PV carriers from the cohort, as measured by AUC. *P*-values are based on the proportion of samples with lower AUC after removing PV carriers from 10,000 bootstrapped samples of the dataset. AUC delta refers to the relative difference in AUC after removing PV carriers in the original cohort

Given the detected interaction of PRS with rare pathogenic variants and age at diagnosis, we next investigated whether the performance of PRS is improved when PV carriers were removed from the cohort. We first stratified the cohort by PRS deciles and observed that compared to those in the middle deciles (Q2–Q9), patients in the top PRS decile are 5.53 (95% CI 1.76–17.97, *p* = 0.003) times more likely to have a first-degree relative with breast cancer (Fig. 7a). Upon removing PV carriers from the cohort, the bottom decile of PRS was depleted of any patients with first-degree relatives with breast cancer, and those in the top decile are now 7.34 (95% CI 2.04–26.66, *p* = 0.002) times more likely to have a first-degree relative with breast cancer compared to those in the middle deciles (Fig. 7b). A similar trend was seen for other groups, where the odds ratio of the top decile group having a family member with breast or other cancer compared to lower decile groups increased upon removing PV carriers. To systematically assess the impact of removing pathogenic variants from our cohort on the performance of PRS AUC, we reevaluated the performance of PRS at detecting relatives with breast cancer using 10,000 bootstrapped samples of the cohort. We calculated the *p*-value as the proportion of samples in which the AUC was lower after removing PV carriers and found that the removal of PV carriers leads to statistically significant (*P* < 0.05) increases in AUC performance across tasks (Fig. 7c). In particular, the performance increased the most in detecting first-degree relatives with breast cancer where the difference in AUC was 0.11, or a 16.5% relative increase in AUC_Family_ after the removal of PV carriers (Additional File 1: Supplementary Table S6, Fig. 7c).

## Discussion

Breast cancer is a significant global health burden that affects millions of people worldwide. While our understanding of breast cancer genetics has improved, leading to changes in screening and intervention strategies, the benefits of these advancements have not been evenly distributed, particularly among understudied populations where the architecture of rare and common germline genetic determinants of the disease remains largely unexplored. In this multicenter study, we made progress toward characterizing the common variant landscape of indigenous Arab breast cancer patients and addressed disparities in genetics research in four ways. First, we demonstrated that lpWGS can be utilized to impute high-quality population-specific variants for the indigenous Arab population. Compared to high-coverage WGS, lpWGS is a cost-effective option both in storage and computation, which makes it a viable option for rapidly increasing data collection in populations where genotype data is scarce and budget may be a significant constraint. As WGS methods continue to improve, existing analysis pipelines on high-coverage WGS may eventually be applied to lpWGS data without substantial loss of power. Second, we have identified an effective ancestry-adjustment method for the calculation of PRS in the Arab population and demonstrated its effectiveness by evaluating its sensitivity at detecting familial breast cancer burden and comparing PRS performance differences before and after the ancestry adjustment. This could increase the power of PRS association studies in understudied populations and serve as a quality-control step for investigating the transferability of existing PRS weights across ancestries. Third, we found individuals with first-degree relatives with breast cancer have complementary PRS distributions based on PV carrier status, providing evidence for the genetic liability threshold model of breast cancer where the threshold for breast cancer may be achieved through a combination of rare or common variant risk [43, 44]. This could have implications for current genetic screening guidelines, as individuals who qualify for genetic screening through a family history of breast cancer but receive negative results from targeted gene panels may now have an additional way of assessing their genetic risks through PRS as more progress is being made toward incorporating PRS into clinical genetics testing practices [31, 45]. Moreover, we showed rare variant risk and common variant risk had distinct roles in contributing to the earlier age of onset among Arab breast cancer patients. While previous studies have also pointed to similar findings where PRS modify the risk for early-onset breast cancer [46–48], most were European-based and did not account for pathogenic variant carrier status or did not quantify the effect of common variant risk on the number of years in earlier onset. To the best of our knowledge, this is the first time a similar observation is made in the Arab breast cancer population with such a large effect size independent of PV carrier status. These observations may elucidate the best subgroup for which PRS testing may yield clinically relevant results and refine Arab population-specific risk assessment of breast cancer. For future studies with sufficient sample sizes and power, focusing on identifying common variants associated with the age of onset among Arab breast cancer patients may be a promising direction to take to uncover the biological mechanism explaining early-onset breast cancers in the indigenous Arab population. Finally, we showed the performance of PRS can be increased by accounting for rare pathogenic variant carrier status among patients. For other studies that may want to investigate the interactions between PRS and other clinical variables, especially in low-sample settings such as those from understudied populations, this may be a useful strategy to employ to increase the power in detecting biological signals.

As our understanding of how polygenic risk may affect breast cancer presentation expands, research focusing on incorporating such information in diverse populations is becoming increasingly important. In this study, we have shown that a PRS that performs the best in detecting breast risk from the general population may not also be the best PRS at predicting familial breast cancer burden. In addition, some PRS have decreased performance when adjusted for the population while some improved. Understanding how to create PRS that is robust to population adjustment will be crucial to creating a PRS that is generally applicable to diverse populations. Moreover, while many methods are being developed to ensure PRS has comparable risk prediction performance across ancestry [49], few have looked at the interactions between PRS with other clinical variables, especially in the context of understudied populations, which could be a missed opportunity to understand how ancestry-specific polygenic risk may affect disease presentation. Compared to cancer-predisposition variants that are under strong selection pressure, variants that do not affect fitness until certain phenotypes develop have less selection pressure and as a result they may vary significantly across ancestries. By evaluating both the risk prediction capability of PRS and its ability to establish clinical correlations, we can reduce the likelihood of a “secondary disparity” scenario whereby even though a developed PRS can predict risk well across ancestries, it is unable to provide further clinical values such as predicting prognosis or responses to therapeutics in non-Europeans. Overall, understanding the potential utility of PRS in understudied populations is important to both addressing existing health inequalities and revealing novel biological insights.

This study has several limitations. First, due to the limited number of publicly available genome-wide variant calls from individuals of Middle Eastern ancestry, the quality of the PRS is assessed indirectly using family cancer history instead of a case-control analysis. Second, while the imputation quality was satisfactory, due to the lack of publicly available servers for lpWGS imputation, we opted to use 1KG as the reference panel as it is more computationally manageable. A reference panel with a larger number of population-specific reference samples could further increase imputation quality. Third, the imputation performance was evaluated based on exome-only, and we assume the imputation performance would be similar in non-coding regions, which may not necessarily hold. Fourth, while this is one of the largest studies that investigated the polygenic risk of breast cancer in the GME region, the sample size is still relatively small compared to studies conducted on European populations, which may underpower our analysis. Fifth, most patients did not have complete clinical data and we were unable to perform survival analysis based on our findings, which may further expand on the utility of PRS. Sixth, while we demonstrated the ability of a previously reported workflow at constructing a PRS capable of detecting hereditary risk, other PRS adaptation methods specialized in generalizing to diverse populations may further improve performance. Finally, family histories of cancer are rarely fully reported, so some individuals with a negative family history may in fact have a family cancer history, further underpowering our analysis .

## Conclusions

Our multicenter observational analysis of 215 lpWGS samples from unrelated breast cancer patients identified a set of biologically plausible PRS that can detect breast cancer burden in the indigenous Arab population. We observed two distinct modes of hereditary breast cancer risk transmission through rare and common variants burden, and found PRS as a potential factor for the early age of onset among indigenous Arab breast cancer patients. We urge for the expansion of genomic studies to include more diverse populations for a comprehensive understanding of hereditary breast cancer risk and call for PRS analysis in the context of the patients.

### Supplementary Information

**Additional file 1 **Contains Supplementary Tables S1 (Clinical characteristics by family history), S2 (Imputation performance by imputation quality scores), S3 (Imputation performance by variant MAF), S4 (PRS performance), S5 (Pathogenic variant carriers and their variant type), and S6 (Effect of removing pathogenic variant carriers on AUC).

**Additional file 2 **Contains Supplementary Figures Fig S1 (ROC curve of age as a predictor of family breast cancer history), Fig S2 (Number of pathogenic variant carriers in known cancer-predisposition genes), and Fig S3 (Age distributions between rare pathogenic variant carriers and non-carriers).

## Data Availability

Source code used for data analysis and visualization as well as anonymized clinical metadata are available on Github [50]. All tools used in this study are publicly available. GLIMPSE is available at the project github page [51]. PRS weights can be accessed on CancerPRSWeb [32]. The docker image containing DeepVariant is available at “gcr.io/deepvariant-docker/deepvariant:1.0.0” and the 1KG calls can be accessed through the google cloud bucket listed in the study: https://console.cloud.google.com/storage/browser/brain-genomics-public/research/cohort/1KGP/cohort_dv_glnexus_opt/v3. For patient privacy and to comply with local regulations, access to the raw sequencing data and variant calls analyzed during the current study are controlled and can be made available by the corresponding author [S.H.A] upon reasonable request. Please allow up to 3 months from request to data sharing to allow enough time to satisfy all regulatory requirements.
