# Supplementary material for: Interplay of Mendelian and polygenic risk factors in Arab breast cancer patients
Source: Genome Med. 2023 Sep 1;15:65. doi: 10.1186/s13073-023-01220-4 (PMC10474689; doi:10.1186/s13073-023-01220-4)
Supplement: Supplementary file 2 — Additional file 2 Contains Supplementary Figures Fig S1 (ROC curve of age as a predictor of family breast cancer history), Fig S2 (Number of pathogenic variant carriers in known cancer-predisposition genes), and Fig S3 (Age distributions between rare pathogenic variant carriers and non-carriers). [file 13073_2023_1220_MOESM2_ESM.docx]

Supplementary Figures

##
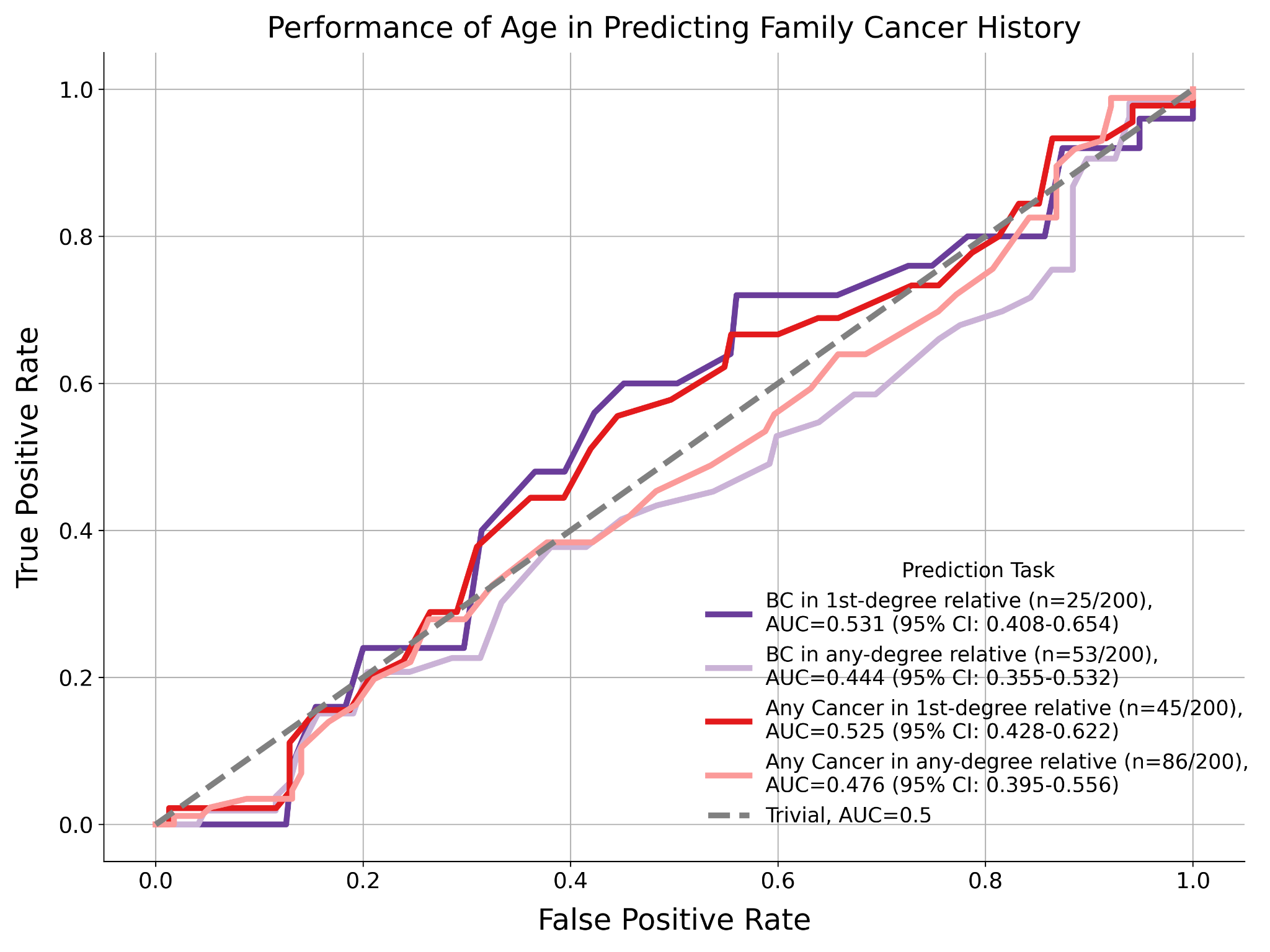


*Figure S1: The AUC curve of using age as a predictor of family breast cancer history. As expected from a negative control, age as a predictor achieves poor performance and the relative ranking of performances is not consistent*

*
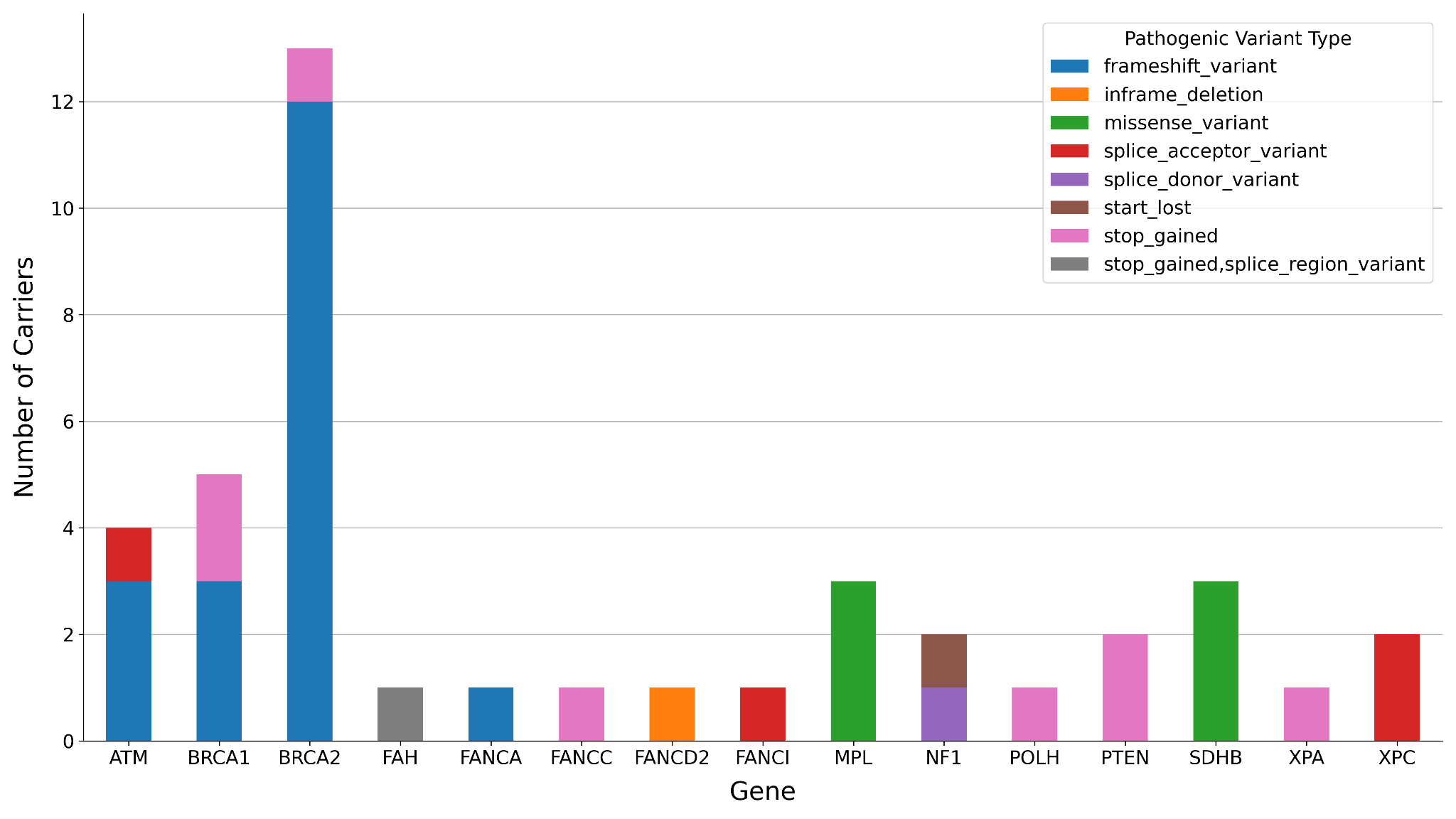
*

*Figure S2. The number of pathogenic variant carriers (n=34) in known cancer-predisposition genes as defined by COSMIC (germline tier 1 genes) and the type of pathogenic variants.*

*
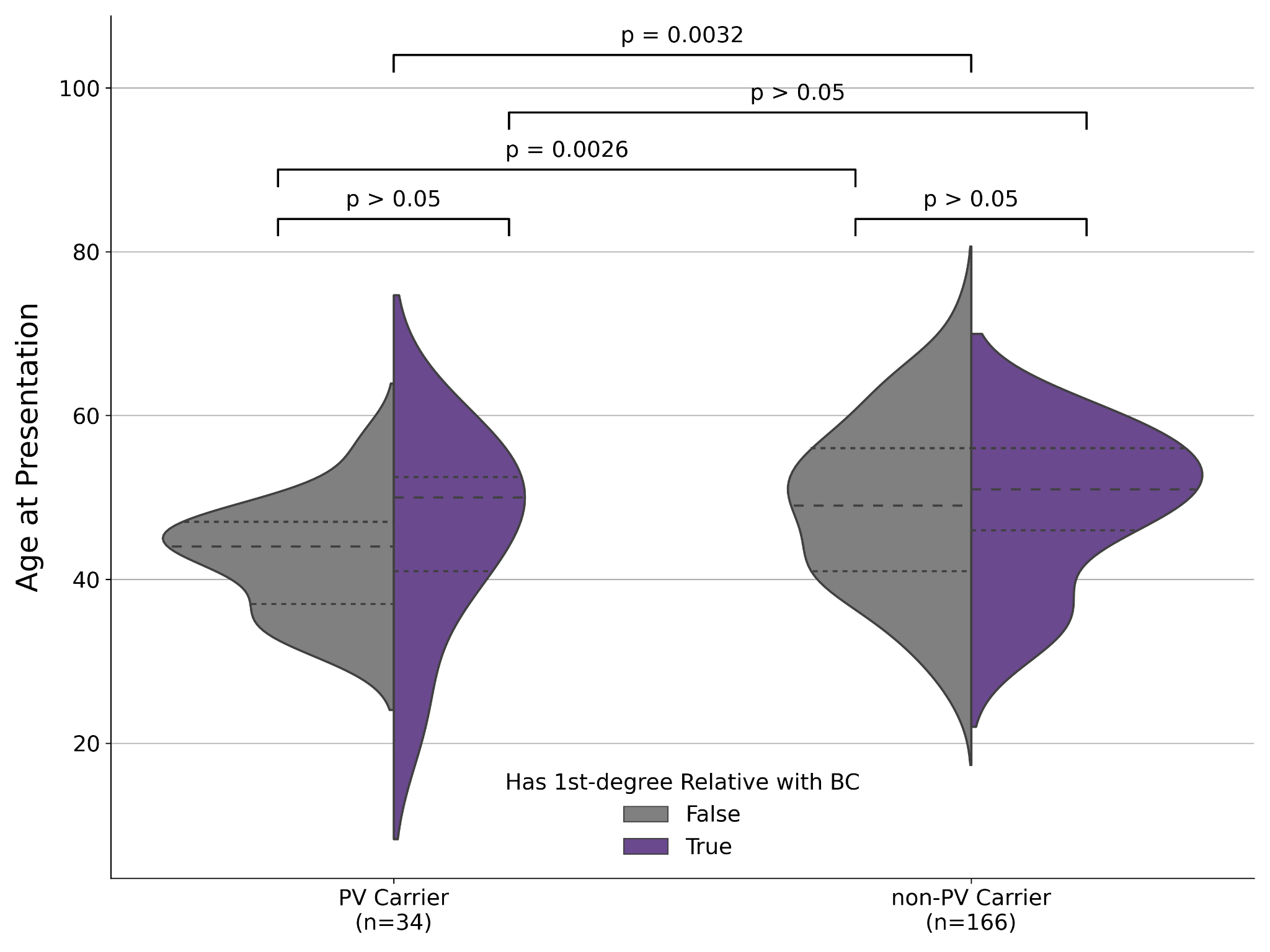
*

*Figure S3. Violin plot of the distributions of age of onset between patients carrying rare pathogenic variants, stratified by whether the patient has a first-degree relative diagnosed with breast cancer (Patients with no first-degree relative with breast cancer: n=175, PV carriers: n=27, non-PV carriers: n=148. Patients with first-degree relatives with breast cancer: n=25, PV carriers: n=7, non-PV carriers: n=18). The dotted line indicates the first and third quartiles and the dashed line indicates the median*
